# Editorial: Advances in drug-induced diseases

**DOI:** 10.3389/fphar.2023.1193099

**Published:** 2023-04-11

**Authors:** Yao Liu, Jia-bo Wang, Linan Zeng, Maxine Gossell-Williams, Patricia Moriel, Miao Yan

**Affiliations:** ^1^ Department of Pharmacy, Daping Hospital, Army Medical University, Chongqing, China; ^2^ School of Traditional Chinese Medicine, Capital Medical University, Beijing, China; ^3^ Department of Pharmacy, West China Second University Hospital, Sichuan University, Chengdu, China; ^4^ Department of Health Research Methods, Evidence, and Impact (HEI), McMaster University, Hamilton, ON, Canada; ^5^ Section of Pharmacology and Pharmacy, Faculty of Medical Sciences Teaching and Research Complex, University of the West Indies, Kingston, Jamaica; ^6^ Faculty of Pharmaceutical Sciences, University of Campinas, Campinas, Brazil; ^7^ Department of Pharmacy, The Second Xiangya Hospital of Central South University, Changsha, Hunan, China; ^8^ Hunan Provincial Center for Poison Information and Treatment, Changsha, Hunan, China; ^9^ International Research Center for Precision Medicine, Transformative Technology and Software Services, Changsha, Hunan, China

**Keywords:** drug-induced diseases, drug safety, FAERS, adverse drug reactions, Editorial

## Introduction

Pathological damage to normal organs or tissues caused by drugs is called drug-induced disease (Wei et al., 2021). In recent years, drug-induced diseases have increased significantly and become a major global public health problem. At the second Global Ministerial Summit on Patient Safety held in Bonn, Germany, in 2017, the World Health Organization (WHO) announced its third global patient safety challenge on medication safety, which aims to reduce serious, avoidable drug-related harm by 50% globally over the next 5 years (WHO, 2017).

Strict drug safety evaluation has been carried out for new drugs before marketing, but some rare and serious adverse drug reactions (ADRs) may occur when the drug is exposed to a large number of people and used for a long time after marketing (Leslie and Schousboe, 2019). Most countries have established pharmacovigilance systems to reduce or avoid drug damage. However, due to factors such as low spontaneous reporting rate, low reporting quality and imperfect system, indeed drug safety data are available, but improvements are needed, and the incidence of drug-induced diseases is seriously underestimated (Pitts et al., 2016; Andrade et al., 2019). In addition, the different intervention and prevention efforts to reduce drug-induced diseases in various countries will lead to significant differences in the outcome of drug-induced diseases. Therefore, drug-induced diseases need more attention and research. The purpose of this Research Topic is to discuss the latest progress of drug-induced diseases and related research, with a view to further understanding and exploring new strategies for prevention and treatment of drug-induced diseases, so as to provide reference for improving drug safety of patients.

This Research Topic contains 19 manuscripts, including six review articles and thirteen original research articles, which extensively discuss the causes of drug-induced diseases, such as drug interactions, drug metabolism, drug transport, and genetic differences between individuals. In addition, this Research Topic aims to get further insights into epidemiology, pathogenic factors, and pathogenesis of drug-induced diseases. We hope to help the authorities formulate policies for the prevention and treatment of drug-induced diseases by exploring new methods to promote the safety of drug use for patients.

Firstly, some cross-sectional studies discussed the risk factors of drug-induced diseases. Huo et al. calculated the linezolid (LZD) exposure using the population pharmacokinetics model of pediatric LZD. And they found that the hematological indexes should be carefully monitored during the treatment by LZD, especially the most common adverse reactions, including thrombocytopenia and low hemoglobin, providing a reference for the personalized drug treatment and clinical treatment risk assessment of LZD. A cross-sectional study in China (Zhao et al.) showed that potentially inappropriate drug (PIM) use in older outpatients with dementia was highly prevalent. Age>80 years, female sex, and taking multiple drugs were risk factors for an increased number of PIM. Xiong et al. found that female sex and cholestatic liver damage pattern were dominant in elderly patients with drug-induced liver injury (DILI) through a retrospective hospitalization-based cross-sectional study. Comorbidities were not the direct factors leading to the severity of DILI. On the contrary, autoimmunity can promote the disease progression of elderly patients with DILI, deserving more intensive treatment and monitoring. In addition, Li et al. conducted a retrospective cohort study. The result indicated that the pharmacist active consultation service could help patients with DILI to obtain better medical care and improve patient outcomes. Hence, they call on pharmacists to participate more in patient care.

Then, the safety of drugs after marketing was analyzed and evaluated through the FDA adverse event reporting system (FAERS). Wan et al. analyzed the safety signal of Bruton’s tyrosine kinase inhibitors and found that patients treated with ibrutinib were more prone to develop adverse events than those treated with acalabrutinib. Jiang et al. summarized the different safety profiles of immunomodulators in multiple myeloma. The results provided a rationale for clinicians and pharmacists to choose suitable immunomodulators for various patients. Xia et al. conducted a pharmacovigilance study that found that antibody-drug conjugates may increase the risk of sepsis in cancer patients, resulting in high mortality. Ma et al. conducted a pharmacovigilance study showing that ocular adverse events associated with anti-VEGF drugs vary. And the results can provide a reference for clinical drug selection.

Thirdly, some literature analyses and reviews have summarized the clinical characteristics of drug-induced diseases. Wang et al. summarized the clinical characteristics of hepatotoxicity of rare ADRs caused by metformin through literature analysis, which is conducive to the diagnosis and timely treatment of hepatotoxicity caused by metformin. Chen et al. retrospectively analyzed the clinical characteristics of mesalazine induced cardiotoxicity through literature, providing basis for clinical diagnosis, treatment and prevention. Wang et al. retrospectively analyzed the clinical features of amoxicillin induced Kounis syndrome (KS), suggesting that amoxicillin induced KS should be considered when chest pain is accompanied by allergic symptoms, electrocardiogram changes and/or elevated levels of myocardial injury markers. Li et al. reviewed the common adverse reactions of CAR-T cell therapy, as well as the mechanism, risk factors, diagnostic criteria and treatment methods of these adverse reactions, providing valuable reference for the safe, effective and wide application of CAR-T therapy. Other meta-analyses evaluate the efficacy and safety of drugs. Wang et al. evaluated chemotherapy-induced peripheral neuropathy (CIPN), and discussed the differences in the efficacy of related therapeutic drugs. The results showed that duloxetine, venlafaxine, pregabalin, crocin, tetrodotoxin, and monosialotetrahexosyl ganglioside might be beneficial to the treatment of CIPN. Luo et al. through systematic review and meta-analysis, found that in patients with coronary heart disease, the co-use of proton pump inhibitors with aspirin and clopidogrel was associated with a reduced risk of gastrointestinal complications, but may increase the incidence of major adverse cardiovascular events, myocardial infarction, stroke, revascularization, and stent thrombosis. Guan et al. compared the cardiovascular safety difference between febuxostat and allopurinol in gout patients through meta-analysis. It was found that febuxostat may have similar cardiovascular characteristics to allopurinol in patients without atherosclerotic disease. However, allopurinol treatment was associated with lower cardiovascular mortality compared with febuxostat in patients with a history of cardiovascular disease.

In addition, Liu et al. successfully established a population pharmacokinetics model of escitalopram and formulated an individualization of dosing regimens based on the age of the patients, CYP2C19 genotype, and serum drug concentrations. The results emphasized that gene detection and therapeutic drug concentration monitoring during treatment were necessary to achieve dosage regimen individualization. Zhang et al. overviewed how overdosage and irrational usage of Euodiae Fructus can induce cardiac side effects at macroscopic and microscopic levels through *in vivo* and *in vitro* experiments and untargeted metabolomics research, providing evidence and reference for the safety research of herbal medicine.

Meanwhile, in our Research Topic, there are two manuscripts discussing drug-induced liver injury and immune-mediated hepatitis (IMH) respectively. Li et al. reviewed the incidence of abnormal liver function in patients with COVID-19 caused by many antiviral drugs, such as favipiravir, remdesivir, lopinavir/ritonavir, and hydroxychloroquine. At the same time, they expounded the possible basic mechanism, and finally put forward reasonable clinical treatment suggestions for such liver injury. Liu et al. introduced in detail the pathophysiology, epidemiology, diagnosis, treatment, and prognosis of IMH caused by immune checkpoint inhibitors.

In conclusion, the Research Topic provides the theoretical basis for the current research on drug-induced diseases to improve the level of clinical medication, ensure the maximum benefit of patients while reducing drug damage, and achieve the goal of rational drug use.
